# Iron parameters analysis in dogs with myxomatous mitral valve disease

**DOI:** 10.1186/s12917-024-04071-2

**Published:** 2024-05-18

**Authors:** Ewa Kumiega, Kamil A Kobak, Agnieszka Noszczyk-Nowak, Monika Kasztura

**Affiliations:** 1 Department of Internal Medicine and Clinic of Diseases of Horses, Dogs and Cats, Faculty of Veterinary Medicine, Wrocław University of Environmental and Life Sciences, Grunwaldzki sq 47, Wrocław, 50-366 Poland; 2 Aging and Metabolism Research Program, Oklahoma Medical Research Foundation, 825 NE 13th St, Oklahoma City, OK 73104 USA; 3 Department of Food Hygiene and Consumer Health Protection, Wrocław University of Environmental and Life Sciences, Norwida St. 25, Wrocław, 50-366 Poland

**Keywords:** Iron status, Myxomatous mitral valve disease, Heart failure, Dogs

## Abstract

**Background:**

Myxomatous mitral valve disease (MMVD) is the most common acquired cardiovascular disease in small breed dogs. In contrast to human patients with heart failure (HF), iron deficiency (ID) prevalence in dogs with MMVD is weakly known. The study aimed to assess the usability of ID markers in serum and reticulocyte parameters from whole blood of dogs with MMVD to evaluate early ID symptoms.

**Results:**

Sixty-eight dogs (43 male and 25 female) were included in the study. MMVD dogs were assigned according to the 2019 ACVIM guidelines for groups B1 (*n* = 9), B2 (*n* = 10), C (*n* = 27) and D (*n* = 10). Groups were also combined into B1 and B2 as non-symptomatic HF and C with D as symptomatic HF. Healthy controls were 12 dogs. Serum iron concentration below the reference range in dogs with MMVD was 12.5%. Other ID indices, such as %SAT, UIBC, and TIBC were similar in the MMVD groups and healthy controls (*p* > 0.05 for all parameters). Statistical comparison between control group and 4 groups of different stages of MMVD showed that significant differences occur only in serum transferrin. The assessment of ferritin and soluble transferrin receptors using Western Blotting did not show differences between control (*n* = 7) and MMVD (*n* = 33) dogs. Study has shown positive correlation between ID parameters and echocardiographic indices such as LA/Ao and LVIDdN, and some biochemical parameters. A significant increase in reticulocytes percentage, assessed manually, was observed in the HF group of animals (*p* = 0.027) compared to the control group.

**Conclusions:**

Studies have shown that ID parameters in serum are not significantly different in dogs with MMVD compared to healthy dogs. However, there is a clear correlation between atrial size and normalised left ventricular size to body size and some biochemical parameters, including ID parameters and therefore the severity of MMVD.

**Supplementary Information:**

The online version contains supplementary material available at 10.1186/s12917-024-04071-2.

## Background

Myxomatous mitral valve disease (MMVD) is dogs’ most common acquired cardiovascular disease and accounts for approximately 75% of chronic heart failure cases [1, 2]. Approximately 30% of dogs over 10 years of age exhibit the characteristic left-sided systolic murmur. MMVD is associated with macroscopic lesions on the mitral and tricuspid valves, leading to poor mitral valve coaptation, insufficiency, and eventually congestive heart failure development [3]. MMVD is characterised by a long asymptomatic period progressing later into a clinical *stadium*, which is observed only in some dogs [1]. The dog owners usually report exercise intolerance, increasing intensity of coughing, and in extreme cases, severe dyspnea as a manifestation of acute pulmonary edema [4]. The high prevalence of iron deficiency (ID) in humans and other species (e.g., rats, mice, and pigs) with heart failure (HF) and its association with worse prognosis make iron deficiency an interesting target in dogs with MMVD. In murine and porcine animal models of HF, ID is associated with exercise intolerance, worsened quality of life, and cardiac remodelling [5–7]. Early indices of anemia, visible as an increased percentage of poikilocytes in the erythrocytes population during detailed assessment of the size and shape changes of red blood cells in blood smear were observed in dogs with MMVD [8]. Although the other red cell parameters obtained from hematological analysers were in the reference values [8]. Iron deficiency (ID) with or without concomitant anemia is one of the frequent comorbidities of HF in humans [9–12] contributing to unfavourable prognosis and poor clinical outcomes. In 2021, the European Society of Cardiology added ID treatment to HF guidelines because of its long-term benefits for patients [13]. According to the guidelines, it is recommended to consider intravenous iron supplementation with ferric carboxymaltose in symptomatic patients with left ventricle ejection fraction (LVEF) < 45% and iron deficiency [13]. Although ID in HF was deeply investigated in recent years [14–16], the mechanisms underlying this condition have not been fully elucidated.

In humans, the routine laboratory diagnosis of ID is based on plasma/serum markers of iron metabolism such as ferritin, transferrin saturation (%SAT), soluble transferrin receptor (sTfR), and hepcidin [11]. Some iron status markers, such as ferritin, may be modified by low-grade inflammation. Nevertheless, these parameters are still useful tools for iron deficiency clinical determination. Basic veterinary laboratory diagnostics usually use a smaller iron diagnostic panel including serum iron and Total Iron Binding Capacity (TIBC). In addition, iron deficiency is usually associated with the presence of anemia.

The presented study aimed to (i) assess the usefulness of ‘traditional’ markers of ID in serum such as iron concentration, ferritin, transferrin, transferrin saturation, soluble transferrin receptor, total binding iron capacity and unsaturated iron binding capacity in dogs with MMVD and (ii) reticulocytes count from whole blood obtained using different laboratory techniques such as, flow cytometry, and manual smear in determining early iron deficiency symptoms, and to (iii) combine raw data obtained from different laboratory analyses to determine discrete changes in iron metabolism in dogs in different stage of MMVD.

## Results

Sixty-eight dogs (43 male and 25 female) were included in the study. None of the dogs from the control (A), B1, B2, C and D groups were classified as chronic kidney disease (CKD) patients as no clinical symptoms of CKD were observed, and both urea and creatinine parameters were in the reference range [17] (Table 1). Also, none of the dogs qualified for the study suffered from liver diseases, as confirmed by the clinical blood analysis and abdomen ultrasonography examination. All animals with abnormalities in their hematological parameters or serum biochemistry (up to a 2-fold increase in the reference range values of aminotransferase activity and urea was considered acceptable) and clinical, ultrasound, or X-ray signs of any other disease apart from chronic MMVD were excluded from the study. In addition, animals clearly skinny or obese, suspected of hyperthyroidism (serum thyroxine concentration > 4.5 µg/dL), diabetes mellitus (serum glucose concentration > 6.7 mmol/L) or oncological diseases were excluded from the study.

**Table 1:** Comparison of echocardiographic, hematological, and iron metabolism parameters in the control group and MMVD dogs

Parameter	*n*	Control group	*n*	MMVD group	*p*
Age [years]	10	8.3 ± 3.0	55	11.0 ± 2.1	**0.0003**
Non-HF/HF (n/n)	-	-	56	(19/37)	-
**Echocardiographic parameters**					
LA/Ao	12	1.33 ± 0.15	55	2.09 ± 0.57	**< 0.0001**
LVIDdN	12	1.54 ± 0.13	54	1.99 ± 0.33	**< 0.0001**
HR [1/min]	10	111 (104; 23)	29	127 (115;170)	**0.011**
**Iron metabolism parameters and reticulocytes**					
Fe [µmol/L]	11	26.8 ± 4.54	56	23.7 ± 7.82	0.21
Transferrin [ng/mL]	12	409.7 (394.7; 447.3)	56	363.6 (312.7; 427.9)	0.14
%SAT	11	29.4 ± 7.5	53	27.6 ± 9.2	0.53
UIBC [µg/dL]	12	362 ± 92	53	357 ± 93	0.86
TIBC[µg/dL]	11	521 ± 78	53	490 ± 98	0.32
Ret - FC [K/µL]	10	59.2 (49.5; 96.7)	54	71.3 (53.4; 99.7)	0.38
Ret -MC [%]	5	0.4 (0.2; 0.5)	53	0.7 (0.4; 1.0)	**0.027**
**Other parameters**					
AST [U/L]	12	26.0 (22.8; 31.3)	56	26.0 (22.0; 33.0)	0.98
ALT [U/L]	12	33.0 (29.0; 57.3)	56	66.0 (47.0; 109.0)	**0.0018**
Urea [mmol/L]	12	5.65 (4.25; 7.00)	56	7.15 (5.50; 9.15)	**0.044**
Creatinine [µmol/L]	12	91.5 ± 25.1	56	85.7 ± 33.1	0.57
Total protein [g/L]	12	60.8 ± 5.7	56	61.4 ± 5.9	0.76
Albumin [g/L]	12	30.5 (28.3; 31.8)	56	32.0 (30.0; 34.0)	0.089
Na^+^ [mmol/L]	11	147 ± 3	55	144 ± 3	**0.0082**
K^+^ [mmol/L]	11	4.51 (4.33; 4.58)	55	4.48 (4.25; 4.82)	0.58
CRP [mg/L]	10	2.15 (2.00; 2.48)	55	2.60 (2.30; 3.00)	**0.024**

Data are presented as mean ± SD or as mean and minimum; maximum result

Abbreviations: %SAT – percentage transferrin saturation, AST- aspartate aminotransferase, ALT - alanine aminotransferase, CRP - C-reactive protein, Fe - iron concentration, HF - heart failure, HR - heart rate, LA/Ao - left atrium to aorta ratio, LVIDdN – left ventricular internal diameters normalized to body weight, MMVD – myxomatous mitral valve disease, Ret-FC - reticulocytes measured by flow cytometry, Ret-MC - reticulocytes measured by manual method, TIBC – total binding iron capacity, UIBC – unsaturated iron binding capacity

LVIDdN = LVIDd (cm)/weight^0.294 (kg)

### Echocardiographic, iron metabolism parameters and reticulocytes

**Fig. 1:**
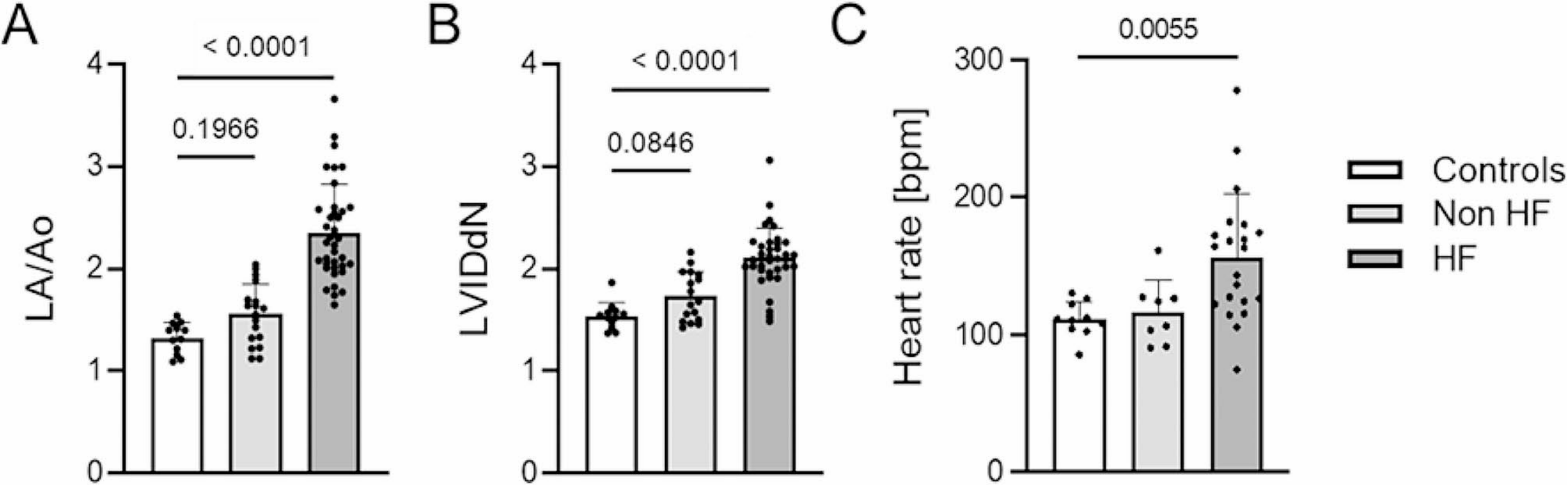
Differences in the left atrium-to-aorta ratio (LA/Ao) (**A**), left ventricular internal diameters normalized to body weight (LVIDdN) (**B**), and heart rate (**C**) between groups of healthy (control), asymptomatic (non HF), and symptomatic (HF) dogs suffering from myxomatous mitral valve disease (MMVD). Exact p-values displayed on graphs. *P* < 0.05 was used to indicate a statistically significant difference

We observed significant differences in echocardiographic parameters such as LA/Lo, LVIDdN, and heart rate between healthy and diseased dogs (B1, B2, C and D) qualified for all MMVD groups (Table 1; Fig. 1, Additional file 1). A significant increase in reticulocytes percentage, assessed manually, was observed in the HF group of animals (*p* = 0.027) compared to the control group.

**Fig. 2:**
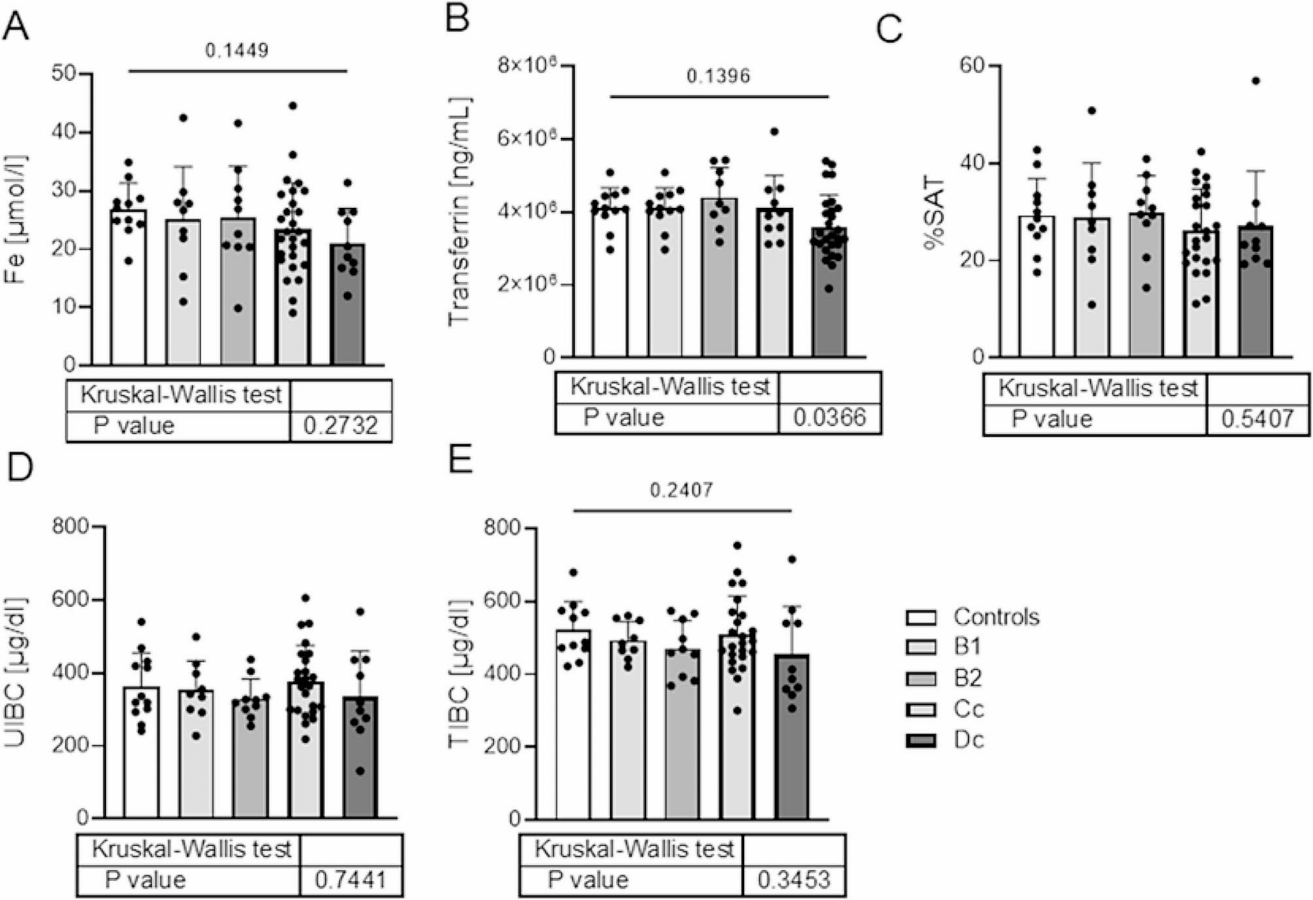
Differences in the ID indices such as iron concentration, transferrin, %SAT, UIBC and TIBC in serum in different stages of MMVD. Exact p-values displayed on graphs. *P* < 0.05 was used to indicate a statistically significant difference

The prevalence of iron deficiency in dogs with MMVD was 12.5% (7 out of 56). Two ID dogs were from the non-HF group, and the remaining 5 belonged to the C (4 out of 27) and D (1 out of 10) groups. Only one dog with an advanced HF had iron deficiency with concomitant anemia. Other ID indices, such as %SAT, UIBC, and TIBC were similar in the control group and MMVD groups (*p* > 0.05 for all parameters; Table 1; Fig. 2).

**Fig. 3:**
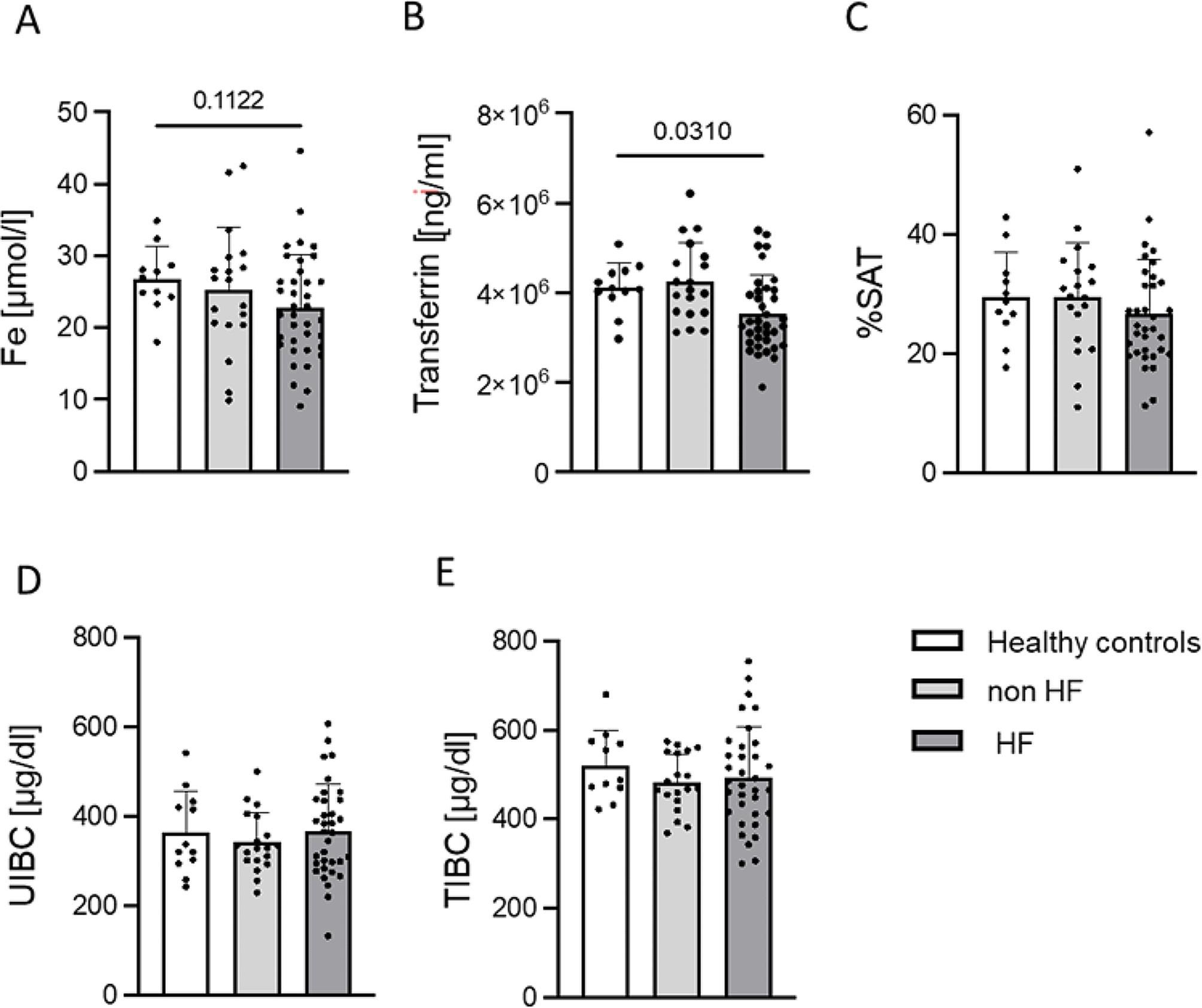
Differences in the ID indices such as iron concentration, transferrin, %SAT, UIBC and TIBC in serum in control group and asymptomatic (non HF; B1 and B2) and symptomatic (HF; C and D) group of MMVD. Exact p-values displayed on graphs. *P* < 0.05 was used to indicate a statistically significant difference

Statistical comparison between control group and 4 groups of different stages of MMVD showed that significant differences occur only in serum transferrin. However, post-hoc analysis did not show any significant difference in pairwise comparisons. Therefore, we combined groups B1 and B2 as non-symptomatic HF and C with D as symptomatic HF to better understand changes occurring in HF dogs. The differences in the values of ID parameters between groups of asymptomatic, symptomatic and healthy control are shown in Fig. 3.

Serum ferritin measured using the ELISA method was undetectable both in the control and MMVD groups of dogs. We did not observe differences between control (*n* = 7) and MMVD (*n* = 33) dogs in ferritin and soluble transferrin receptor measured using Western blotting (Additional file 2).

### Other biochemical parameters

We found significant differences between the control and MMVD group in ALT, urea, Na^+^, and CRP concentrations (*p* < 0.05 for all parameters, Table 1), with higher values in the MMVD group recorded for all mentioned parameters except for Na^+^ concentration. ALT and urea concentrations were only slightly above the reference values (> 100 U/l for ALT and > 8,9 mmol/l for urea). Higher levels of ALT, but not exceeding the 2-fold reference value, were recorded in 7 dogs with concomitant significant tricuspid regurgitation and concomitant symptoms of right ventricular heart failure (5/7 dogs from HF group). Higher CRP values noted for the MMVD dogs did not exceed the reference values (0–5 mg/l).

### Correlations between iron metabolism indices and echocardiographic, and biochemical parameters

UIBC positively correlated with total protein (*R* = 0.44, *p* = 0.001 and albumin (*R* = 0.33, *p* = 0.008). Also TIBC positively correlated with total protein (*R* = 0.43, *p* = 0.001) and albumin (*R* = 0.3, *p* = 0.016).

We observed that the increase in left atrium size (increased LA/Ao ratio) was related to the decrease in iron concentration in dogs serum (*R* = -0.38, *p* = 0.002), transferrin concentration (*R* = -0.35, *p* = 0.004), and %SAT (*R* = -0.32, *p* = 0.011). The left atrium size positively correlated with the absolute reticulocytes count (*R* = 0.25, *p* = 0.045) and reticulocytes percentage (*R* = 0.28, *p* = 0.035) (Table 2).

**Table 2:** Correlations between echocardiographic and reticulocytes parameters in dogs with myxomatous mitral valve disease included in the study

Parameter	Ret [K/µL]	Ret [%]	Ret [%]
	FC	FC	MC
**LA/Ao**	*R* = 0.25 *p* = 0.045		*R* = 0.28 *p* = 0.035
**LVIDdN**	*R* = 0.31 *p* = 0.015	*R* = 0.30 *p* = 0.019	*R* = 0.27 *p* = 0.045

Abbreviations:, FC – flow cytometry, LA/Ao – left atrium-to-aorta ratio, LVIDdN – left ventricular internal diameters normalized to body weight, MC – manual counting, Ret – reticulocytes

**Fig. 4:**
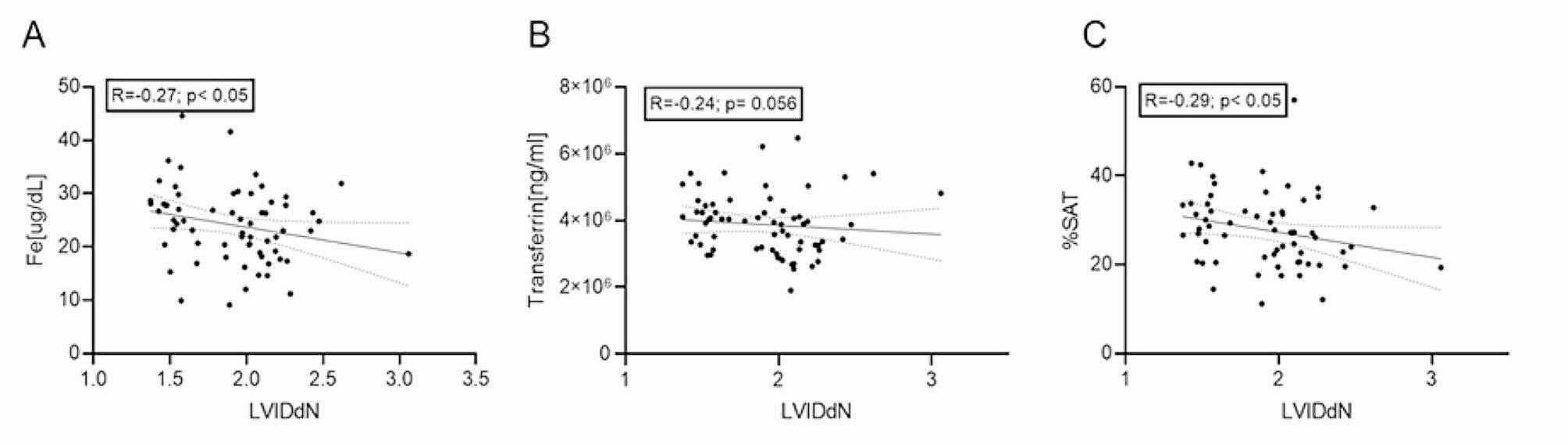
Correlations between left ventricular internal diameters normalized to body weight (LVIDdN) parameter and iron (**A**) and transferrin (**B**) concentration, and transferrin saturation (%SAT) in dogs with myxomatous mitral valve disease (MMVD) included in the study analyzing iron parameters in dogs with various stages of MMVD

Also, we noted that the increase in LVIDdN corresponded to iron concentration (*R* = -0.27, *p* = 0.031) and %SAT (*R* = − 0.29, *p* = 0.024) decrease (Fig. 4). We observed no correlations between UIBC or TIBC and measured echocardiography parameters (Table 3). Additionally, the increase in LVIDdN correlated with absolute reticulocytes count measured automatically (*R* = 0.31, *p* = 0.015) and reticulocytes percentage (*R* = 0.27, *p* = 0.045).

**Table 3:** Correlations between iron metabolism parameters and echocardiographic, hematological and biochemical parameters in dogs with MMVD

Parameter	Fe [µmol/L]	%SAT	UIBC [µg/dL]	TIBC [µg/dL]
**Echocardiography parameters**				
LA/Ao	*R* = -0.38 *p* = 0.002	*R* = -0.32 *p* = 0.011		
LVIDdN	*R* = -0.27 *p* = 0.031	*R* = -0.29 *p* = 0.024		
**Other biochemical parameters**				
AST [U/L]			*R* = -0.25 *p* = 0.047	*R* = -0.35 *p* = 0.005
Urea [mmol/L]			*R* = -0.32 *p* = 0.001	*R* = -0.42 *p* = 0.001
CRP [mg/L]	*R* = -0.40 *p* = 0.001	*R* = -0.35 *p* = 0.005		
Total protein [g/L]		*R* = -0.26 *p* = 0.040	*R* = 0.44 *p* = 0.001	*R* = 0.43 *p* = 0.001
Albumin [g/L]			*R* = 0.33 *p* = 0.008	*R* = 0.30 *p* = 0.016

Abbreviations: %SAT – percentage transferrin saturation, AST – aspartate aminotransferase, CRP – C-reactive protein, Fe- iron concentration, LA/Ao – left atrium-to-aorta ratio, LVIDdN – left ventricular internal diameters normalized to body weight, MMVD –myxomatous mitral valve disease, TIBC – total binding iron capacity, UIBC – unsaturated iron binding capacity

## Discussion

The relationship between iron deficiency with or without concomitant anemia and human heart failure has been widely researched for many years. It is known that heart performance decrease correlates with a lowered iron concentration in the blood. Hence, iron metabolism is routinely examined, and intravenous supplementation is recommended, e.g. with ferric carboxymaltose, in patients with heart failure [13, 18]. In dogs with heart failure, multi-parameter iron metabolism is not routinely assessed [19]. The exclusion or confirmation of anemia is most often based on the red blood cell parameters and serum total iron concentration assessment.

In our study, we analyzed iron metabolism in 68 dogs using such parameters as serum iron concentration, TIBC, UIBC, %SAT, transferrin, ferritin, and soluble transferrin receptor concentration. The reticulocytes count was measured using an automated method (flow cytometry) and manual method using blood smear assessed.

The study groups differed statistically in terms of age and sex, which agrees with the fact that MMVD is more prevalent in older, over 8–10 years of age, male dogs [20]. However, the study groups did not differ significantly in body weight.

By analyzing the obtained data we observed a higher percentage of reticulocytes in MMVD dogs using the manual method. Similar results were obtained by Choi et al. [21]. The authors observed an increase in reticulocytes in 80.9% of MMVD dogs, which was associated with dyspnea due to cardiogenic pulmonary edema (CPE). Reduced mean erythrocyte volume (MCV) is characteristic of iron deficiency anemia. In the dogs with MMVD we studied, MCV values were within the reference ranges (60–77 fL [17], Additional file 3) but the erythrocyte volume tended to decrease along with HF severity increase. The differences in echocardiographic parameters and HR observed between the control and MMVD groups were related to the MMVD pathophysiology and characteristic of progressive changes eventually leading to HF development.

In the studied MMVD dogs, LVIDdN positively correlated with reticulocytes assessed both with flow cytometry and manually smear, but the increase in left atrium-to-aorta ratio (LA/Ao) positively correlated only with the reticulocytes percentage calculated from manually smear. This means that it can be assumed that the manual method of assessing reticulocytes should be considered for dogs with MMVD.

UIBC, TIBC, and % SAT did not differ significantly between the control and MMVD groups. Similar results were also obtained by Savarese et al. [22] despite a significant percentage of dogs with renal insufficiency included in their study. They found that the decrease in serum iron concentration was more frequent in dogs with heart failure and correlated with LA/Ao. However, the authors underlined that examined parameters of iron metabolism, i.e., serum iron concentration, TIBC, UIBC, and %SAT, and a small group of symptomatic dogs (*n* = 18) may not be sufficient to assess iron deficiency [22]. Due to this reason, in our study, we tried to include an assessment of such parameters as sTfR, ferritin, transferrin and reticulocytes, based on human and animal medicine guidelines for patients with non-cardiovascular diseases [10, 11, 18, 22] to test if the enlarged panel of iron metabolism indices allows predicting the ID more accurately in dogs.

Iron, transferrin concentration, and % SAT negatively correlated with the left ventricular internal diameters normalized to body weight (LVIDdN), showing that the progression of MMVD is accompanied by slowly developing iron deficiency. The obtained results show that the decrease in iron levels can occur without accompanying anemia, but can certainly lead to anemia later. Data presented in our study showed that only 12.5% symptomatic dogs had value of concentration iron in serum below reference range. The RBC count is also not visibly declining and fits within the reference ranges (Additional file 3). We can assume that analyzing only the RBC count and hemoglobin concentration for iron deficiency diagnosing in dogs with heart failure is not fully reliable. The manual smears done in our previous studies confirmed that a more severe erythrocyte anisocytosis is showing in dogs with symptomatic MMVD (groups C and D) compared to the control group [8]. In addition, erythrocyte poikilocytosis appeared more frequently as the heart failure progressed [8].

Iron deficiency always translates into a worse condition of the whole organism and individual cells, especially those with high energy requirements, such as cardiomyocytes or muscle cells [23]. Hence, the iron balance in various human disease stages and safe iron supplementation in different clinical conditions are being studied with increasing attention. It has been observed that in physiological conditions, iron is not excreted from the body, and any iron excess is stored in safe cell deposits in the form of ferritin, distributed mainly in the liver macrophages, spleen, and other organs and cells [7]. In the presented study, Western blotting showed the presence of good quality bands for ferritin and soluble transferrin receptor, which did not differ between the studied groups. It should be noted that we used antibodies specific for human ferritin and transferrin because of a shortage of reliable dog’s specific antibodies.

Iron deficiency may result from the complete depletion of this element in the body (absolute ID) due to the lack of iron in the diet, malabsorption disorders, and strong or frequent hemorrhages [10]. It may also present as a functional ID that is associated with the body’s inability to use its natural iron reserves. The functional ID can be observed in inflammation accompanied by an increase in pro-inflammatory factors, like IL-6 and hepcidin, in liver disorders, and some genetic diseases (hemochromatosis). Persistent inflammation restricts the pool of free circulating iron leading to excessive iron accumulation in ferritin, which can transform into toxic deposits of hemosiderin [10]. Interestingly, in our study, CRP values, although statistically significantly higher in the group of dogs with MMVD, did not exceed the reference values. This may suggest that the subinflammatory state occurring during MMVD is probably not the main cause leading to ID.

The analysis of correlations between iron metabolism and reticulocytes count, blood biochemical, or echocardiography parameters allowed us to assess in detail the relationship between iron concentration in serum and the functioning of the whole organism. Similarly, the observed increase in ALT and urea concentration was also associated with HF development. The observed increase in urea and the decrease in Na^+^ in MMVD dogs most probably result from the treatment based on loop diuretics that dogs with HF symptoms received.

The cause of iron deficiency progression in humans with heart failure is unknown. However, since the prognosis and survival of HF patients with ID (with or without anemia) are worse compared to HF patients without ID, the European Society of Cardiology (ESC) recommends supplementing iron intravenously, e.g., with ferric carboxymaltose, in HF patients with ID [13, 18]. Iron deficiency has been reported in animals (pigs and rodents) with experimentally induced HF [7, 24]. However, only a few studies on iron management in HF dogs are available [22, 25], despite HF being a common disease of older and small breed dogs, especially Cavalier King Charles Spaniel (CKCS), dachshund, miniature poodle, Maltese, Chihuahua, Pomeranian, or Yorkshire terriers [1]. Studies have shown that iron deficiency in HF dogs is less frequently observed than in HF human patients [22]. The presented study shows that iron deficiency in dogs relates to abnormal red cell images observed in manual smears [8], while other classic iron parameters (total iron concentration, %SAT, TIBC, UIBC, sTfR) remain normal. So do dogs differ from other species in their iron metabolism?

There is certainly no clear answer to this question. It is known, however, that the course of HF in dogs is different than in humans or experimental animals. The non-ischemic etiology of HF predominates in dogs. A typical echocardiographic image of heart failure in a small breed dog is volume overload associated with mitral valve insufficiency. The consequence of this is deteriorating exercise tolerance, coughing, and faster respiration. The first clinical symptoms become noticeable and disturb the pet owner relatively long after the start of the heart remodelling process. Left ventricular heart failure due to MMVD is associated with the dilatation of the left atrium and the left ventricle, with the systolic function of the left ventricle preserved. Also, arrhythmias, worsening left ventricular output, are much less common in dogs with MMVD and in more advanced stages of the disease than in dogs with dilated cardiomyopathy or in people with ischemic heart disease [26, 27]. Perhaps these differences influence the different picture of iron metabolism in dogs developing HF in the course of MMVD, which makes observing its disturbances much more difficult.

A major limitation of the study was the lack of commercially available species-specific canine antibodies to ferritin and soluble transferrin receptors we used in the Western blot.

## Conclusion

The analysis of iron balance should be based on a multistage biochemical study, including serum iron and transferrin concentration and transferrin saturation (%SAT). The analysis should be complemented by ferritin and sTfR levels. However, a reliable methodology is yet to be developed. The differences in standard blood parameters, such as iron concentration, TIBC, UIBC, %SAT, transferrin, and ferritin in serum, between healthy and MMVD dogs are intangible but show a clear correlation with atrial size and normalised left ventricular size to body size and some biochemical parameters and therefore the severity of MMVD.

## Methods

### Groups of animals

A total of 68 dogs (43 males and 25 females) were qualified for the study (Table 4). In our study took part the same dogs like in the studies Kumiega et al. 2020 [8]. The control group (group A) consisted of healthy dogs, 7 male, and 5 female. Animals from the B1 and B2 groups consisted of appropriately 9 and 10 patients with asymptomatic mitral valve insufficiency (non-HF group), whereas patients from C and D groups consisted of 27 and 10 symptomatic individuals (HF group), respectively. Apart from the assigned dogs according to the 2019 ACVIM guidelines [28], in this study we took into account non-symptomatic group (non-HF; B1 and B2) and symptomatic group (HF group; C and D). In our opinion this division would be helpful for clinicians. Many studies chose to combine groups C and D into a group of symptomatic dogs [29] or B1 and B2 as an asymptomatic group [30, 31] for statistical analysis. The results obtained were also analyzed with a division into asymptomatic and symptomatic dogs [32, 33], as in this article.

**Table 4:** Groups of dogs qualified for the study

Group		*n*	Age [years]	Weight [kg]	Sex (*n*)		Breed (*n*)
					male	female	
control	A	12	8.2 ± 3.0	14.0 ± 5.3	5	7	Beagle (5), Border collie (2), Nova Scotia duck tolling retriever (1), miniature schnauzer (1), Cavalier King Charles spaniel (1), mixed-breed (2)
non-HF	B1	9	10.35 ± 2.6	10.1 ± 2.7	8	1	Dachshund (2), miniature schnauzer (2), shih-tzu (1), fox terrier (1), mixed-breed (3)
	B2	10	11.3 ± 2.7	9.1 ± 4.4	6	4	Dachshund (2), miniature schnauzer (1), shih-tzu (1), cocker spaniel (1), Yorkshire terrier (1), Cavalier King Charles spaniel (1), mixed-breed (3)
HF	C	27	11.4 ± 1.9	10.0 ± 5.0	18	9	Miniature schnauzer (4), Cavalier King Charles spaniel (3), shih-tzu (3), dachshund (2), medium poodle (1), Pekingese (1), bull terrier (1), mixed-breed (12)
	D	10	11.8 ± 1.9	8.5 ± 3.7	6	4	Cavalier King Charles Spaniel (1), bull terrier (1), Yorkshire terrier (1), miniature poodle (1), dachshund (1), Pekingese (1), mixed-breed (4)

Data are presented as mean ± SD. Abbreviations: A – control, B1, B2, C, and D – dogs with myxomatous mitral valve disease (MMVD) grouped according to the American College of Veterinary Internal Medicine (ACVIM) consensus guidelines classification for MMVD, non-HF group - asymptomatic group (B1 and B2); HF group - symptomatic group (C and D)

All the dogs were patients of the Department of Internal Medicine with the Clinic of Diseases of Horses, Dogs, and Cats, Faculty of Veterinary Medicine, Wrocław University of Environmental and Life Sciences. Written informed consent was obtained from all the owners.

After a detailed anamnesis, physical examination, a 6-lead ambulatory electrocardiographic examination, transthoracic echocardiography (Aloka F36 or Aloka Arietta V60; Hitachi-Aloka, Tokyo, Japan), and a routine blood evaluation 12 healthy dogs subjected to preventing screening were used as a control group (group A). The remaining dogs with different stages of myxomatous mitral valve disease (MMVD) disease were assigned into asymptomatic dogs: B1, B2 or symptomatic dogs: C and D groups according to the American College of Veterinary Internal Medicine (ACVIM) consensus guidelines classification for MMVD disease. The non-HF group included asymptomatic dogs in the B1 and B2 MMVD stages. The heart failure group included symptomatic dogs in the C and D MMVD stages. Group C included dogs with past clinical signs of heart failure secondary to MMVD. Group D included dogs with end-stage MMVD disease with clinical signs of heart failure resistant to standard pharmacological treatment.

LA/Ao was measured from a right parasternal short-axis view at the heart base. Both linear measurements were made in early diastole, which was defined by the earliest frame in which the closed aortic valve cusps could be visualized as previously described [34]. LVIDD was measured in the right parasternal long axis view in M-mode recordings. Measurements of LVIDd at the beginning of the QRS complex and LVIDs at the end of T wave were made by drawing a line starting from the midpoint of the septal arc to the LV free wall. The measurement was performed 3 times, and the result was averaged. The examination was performed by a specialist with experience in echocardiography.

### Blood collection and reticulocytes analysis

All the animals were fasted for at least 12 h before the examinations. The samples of venous blood from all the studied animals were drawn into serum and ethylenediaminetetraacetic acid (EDTA) tubes. The blood samples collected for serum were stored for 30 min at room temperature, then centrifuged at 4000 g for 5 min, and transferred for biochemical analysis. The remaining serum samples were immediately frozen and stored at -80 °C until further analysis.

Reticulocytes were performed with the automated hematology analyzers using flow cytometry (LaserCyte Dx, IDEXX Laboratories, Westbrook, MN, USA). For microscopic reticulocyte determination, blood collected to EDTA tubes was mixed with a vital stain: 1% new methylene blue with 1.6% potassium oxalate anticoagulant and 1% brilliant cresol blue 1% in saline (ANALAB Sp. Z o.o, Poland). Blood and stain were mixed in equal amounts and incubated for 30 min at room temperature. The mixture smear was air dried, and the percentage of reticulocytes per 1000 non-nucleated RBCs was calculated. The percentage of reticulocytes was counted twice by a laboratory diagnostician. Reticulocyte results were corrected with haematocrit (HCT) according to the formula: Corrected RETIC % = RETIC % × (patient’s HCT/normal HCT) [35].

The percentage of reticulocytes was calculated manually and by an automated method to compare both research techniques and increase the reliability of the results.

### Biochemical and iron metabolism parameters analysis

Serum biochemical panels and iron status parameters were done using an automated analyzer (Konelab Prime 30ISE; Thermo Scientific, Waltham, Massachusetts, USA).

Serum iron concentrations (µmol/L) and unsaturated iron binding capacity (UIBC) (µg/dL) were assessed with a substrate method using Ferene S (Thermo Fisher Scientific, Warsaw, Poland). Total iron binding capacity (TIBC) (µg/dL) was calculated as the sum of UIBC and iron concentration [35]. Transferrin saturation was calculated as a serum iron (µg/dL) and TIBC (µg/dL) ratio and expressed as a percentage (%SAT) [36]. Serum transferrin and ferritin were assessed by ELISA kits (MyBiosource, USA), following the manufacturer’s guidelines.

CRP concentration was determined using a canine-specific immunoturbidimetric method using an automated analyzer (Konelab Prime 30ISE; Thermo Scientific, Waltham, Massachusetts, USA).

In dogs, iron deficiency (ID) is diagnosed < 16.8 µmol/L [17]. The reference range for UIBC was 142–393 [µg/dL], for TIBC 284–537 [µg/dL], and for %SAT 20–59% as proposed by Harvey et al. [37]. Anemia is diagnosed when hemoglobin concentration is < 7.45 mmol/L [17].

### Western blotting of ferritin and Soluble transferrin receptor (sTfR)

As samples containing both ferritin and sTfR were separated using different types of gels for SDS PAGE also samples preparation for ferritin and sTfR were unique we decided to prepare two separately acapits for both protein molecules.

**Ferritin** Ferritin was isolated from serum samples according to a modified procedure by Miyazaki et al. [38, 39]. Briefly, 50 µL of diluted samples were heated to 80 °C for 10 min and then centrifuged for 15 min at 14,000 g at 4 °C. In this procedure, ferritin molecules remain in the solution, whereas other proteins denature and are removed by centrifugation. The ferritin-enriched supernatants (20 µL) were mixed with 5 µL of a non-reducing sample buffer (Pierce, Rockford, USA) and incubated for 10 min at 40 °C. Then, the samples were loaded on 1% SDS-PAGE gels (4% stacking gel and 8% acrylamide separating gel) and wet-transferred to a PVDF membrane (Millipore, Poland). The membrane was treated with the Quentix Signal Enhancer (Pierce, Thermofisher Scientific, Warsaw, Poland), blocked for 1 h with 5% skimmed milk in the PBS containing 0.5% (v/v) Triton X-100 (Sigma-Aldrich, Darmstadt, Germany), and incubated overnight with rabbit antibodies against ferritin heavy chain (1:500) (Abcam, Symbios, Straszyn, Poland) or ferritin light chain (1:5000) (Abcam, Symbios, Straszyn, Poland). Next, membranes were washed 3 × 15 min with 0.5% skimmed milk in the PBS containing 0.5% Triton X-100, treated with HRP-conjugated goat anti-rabbit secondary antibodies (1:40 000) (Jackson Immunoresearch, Poland) for 1 h and washed as above. Bands were detected using the SuperSignal West Femto ECL substrate (Pierce, Thermofisher Scientific, Warsaw, Poland). Bands’ intensity was determined by densitometric scanning using the ChemiDoc MP Imaging System and Image LabTMSoftware v.6.0 (BioRad, Warsaw, Poland). Each sample was analyzed in triplicate.

**Soluble Transferrin Receptor** Serum samples from 40 selected dogs were diluted 5 times with distilled water, and then 20 µL of each sample were mixed with 5 µL of a non-reducing sample buffer (Pierce, USA), incubated for 10 min at 40 °C. Then they were loaded on Criterion TGX stain-free precast 4–15% gradient gel (BioRad, Warsaw, Poland), and wet-transferred on PVDF membrane. The membranes were treated as described for ferritin, and the following antibodies were used for sTfR detection: against sTfR (1 : 500) (Abcam, Symbios, Straszyn, Poland), HRP-conjugated goat anti-rabbit secondary antibodies (1 : 40 000) (Jackson Immunoresearch, Poland).

### Statistical analysis

Most of the continuous variables had a normal distribution and were expressed as a mean ± standard deviation (Me ± SD). Variables with skewed distribution were expressed as a median with an interquartile range (Me (Q1; Q3)). Differences in values between healthy controls and MMVD animals were analyzed using an unpaired Student’ t-test or Mann–Whitney test. Differences in values between 3 groups of animals (healthy controls, non-HF, and HF) were analyzed with the one-way ordinary ANOVA test followed by Dunnett’s multiple comparisons test or Kruskal–Wallis test followed by Dunn’s multiple comparisons test. Spearman’s rank correlation coefficient (R) reflects the relationship between laboratory and echocardiography parameters in all animals. *P* < 0.05 was used to indicate a statistically significant difference. Statistical analyses were performed using GraphPad Prism Software (GraphPad Software Inc., San Diego, CA, USA).

### Electronic supplementary material

Below is the link to the electronic supplementary material.

Supplementary Material 1

Supplementary Material 2

Supplementary Material 3

Supplementary Material 4

## Data Availability

The datasets used and/or analysed during the current study are available from the corresponding author on reasonable request.
