# Supplementary material for: Iron parameters analysis in dogs with myxomatous mitral valve disease
Source: BMC Vet Res. 2024 May 18;20:210. doi: 10.1186/s12917-024-04071-2 (PMC11102178; doi:10.1186/s12917-024-04071-2)
Supplement: Supplementary file 1 — Supplementary Material 1 [file 12917_2024_4071_MOESM1_ESM.docx]

**Additional file 1:** Echocardiographic parameters such as LA/Lo, LVIDdN, and heart rate between healthy and diseased dogs.

**
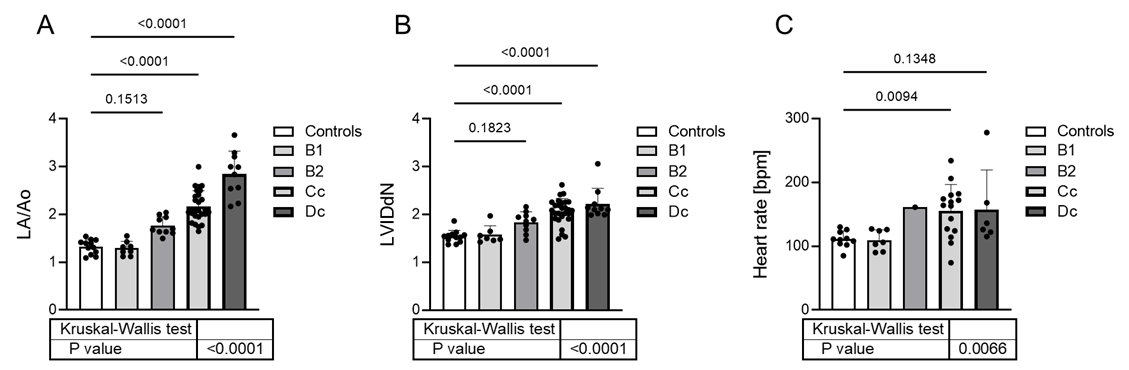
**

Supplementary Figure 1. Differences in the left atrium-to-aorta ratio (LA/Ao) (A), left ventricular internal diameters normalized to body weight (LVIDdN) (B), and heart rate  C) between groups of healthy (control), and group B1, B2, C and D) dogs suffering from myxomatous mitral valve disease (MMVD).
