# Supplementary material for: Iron parameters analysis in dogs with myxomatous mitral valve disease
Source: BMC Vet Res. 2024 May 18;20:210. doi: 10.1186/s12917-024-04071-2 (PMC11102178; doi:10.1186/s12917-024-04071-2)
Supplement: Supplementary file 2 — Supplementary Material 2 [file 12917_2024_4071_MOESM2_ESM.docx]

**Additional file 2.**

Sera from a studied groups of dogs were analyzed by Western blotting using appropriate antibodies. Proteins were assayed in three independent experiments

**
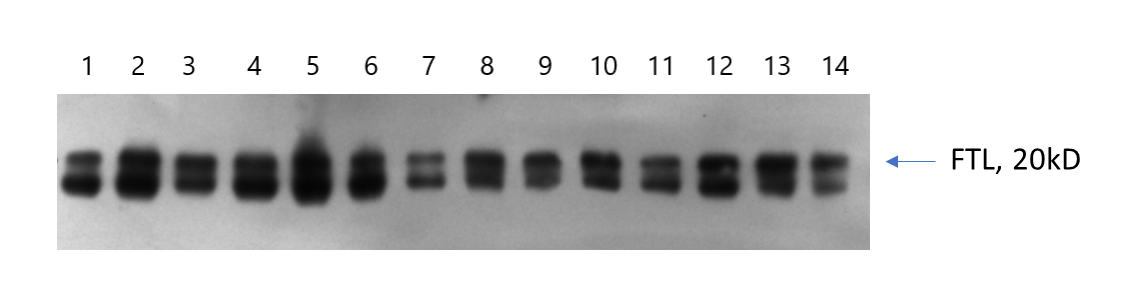
**

Supplementary Figure 1 : FTL - ferritin light chains; 1,2,3 - healthy dogs; 4,5,6 - B1 MMVD dogs; 7,8 - B2 MMVD dogs; 9,10,11 - C MMVD dogs; 12,13,14 - D MMVD dogs.


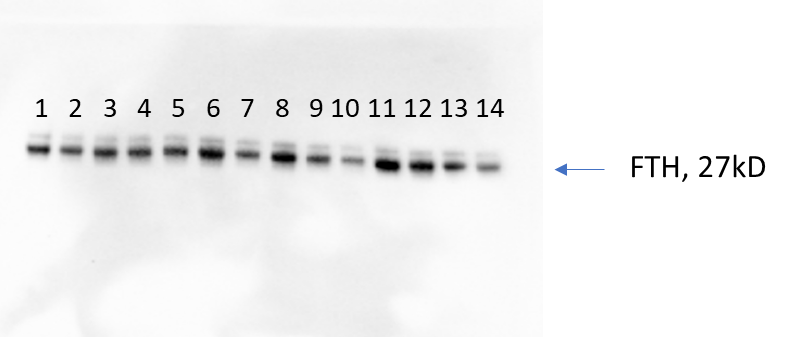


Supplementary Figure 2: FTH- ferritin heavy chains; 1,2,3 – healthy dogs; 4,5,6 – B1 MMVD dogs; 7,8 – B2 MMVD dogs; 9,10,11 – C MMVD dogs; 12,13,14 – D MMVD dogs.


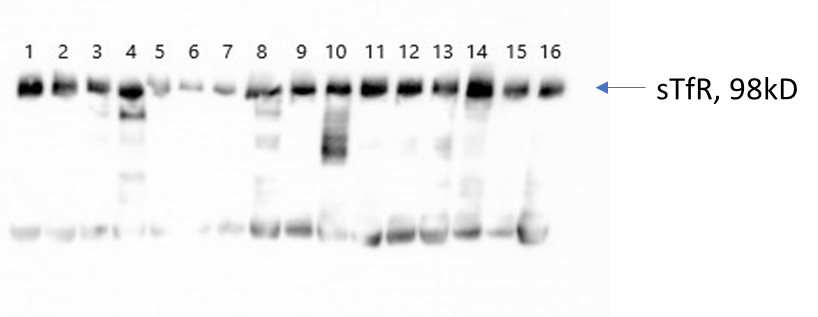


Supplementary Figure 3 : sTfR — transferrin soluble receptor; 1,2,3,4– healthy dogs; 5,6,7 –B1 MMVD dogs; 8,9,10 – B2 MMVD dogs; 11,12, 13 – C MMVD dogs; 14,15,16 – D MMVD dogs.
