# Supplementary material for: Iron parameters analysis in dogs with myxomatous mitral valve disease
Source: BMC Vet Res. 2024 May 18;20:210. doi: 10.1186/s12917-024-04071-2 (PMC11102178; doi:10.1186/s12917-024-04071-2)
Supplement: Supplementary file 3 — Supplementary Material 3 [file 12917_2024_4071_MOESM3_ESM.docx]

**Additional file 3**. Comparison of red blood cell parameters between the control group (A) and groups B1, B2, C and D of dogs with MMVD [8].

|  | Groups | | | | |
| --- | --- | --- | --- | --- | --- |
|  | A | B1 | B2 | C | D |
| RBC  [ M/µl] | 6,86 ± 0,63 | 7,30 ± 0,89 | 7,10 ± 0,90 | 6,99 ± 0,69 | 6,95 ± 1,23 |
| HCT  [%] | 49,04 ± 5,65 | 51,33 ± 5,11 | 50,19 ± 0,10 | 48,56 ± 4,29 | 47,9 ± 7,42 |
| HGB  [mmol/l] | 10,13 ± 0,87 | 10,77 ± 1,08 | 10,34 ± 0,72 | 10,21 ± 0,92 | 10,05 ± 1,30 |
| MCH  [fmol] | 1,47 ± 0,08 | 1,47 ± 0,11 | 1,46 ± 0,12 | 1,46 ± 0,10 | 1,46 ± 0,11 |
| MCHC  [mmol/l] | 20,73 ± 1,21 | 20,99 ± 1,48 | 20,71 ± 1,39 | 20,94 ± 1,20 | 21,23 ± 1,32 |
| MCV  [ fL] | 71,36 ± 2,79 | 70,52 ± 3,72 | 70,74 ± 3,30 | 69,54 ± 4,82 | 68,62 ± 3,70 |
| RDW  [%] | 15,83 ± 0,93 | 15,59 ± 0,58 | 15,27 ± 0,29 | 15,59 ± 0,63 | 15,53 ± 0,41 |

Data are presented as mean ± SD. Abbreviations: HCT- hematocrit, HGB- hemoglobin concentration, MCH - mean corpuscular hemoglobin, MCHC - mean corpuscular hemoglobin concentration, MCV - mean corpuscular volume, RBC - red blood cel, RDW - red cell distribution width.

The analyser: LaserCyte Dx, IDEXX Laboratories, Westbrook, MN, USA.

p > 0.05 for all parameters.
