# Supplementary figures and images for: Iron parameters analysis in dogs with myxomatous mitral valve disease
Source: BMC Vet Res. 2024 May 18;20:210. doi: 10.1186/s12917-024-04071-2 (PMC11102178; doi:10.1186/s12917-024-04071-2)

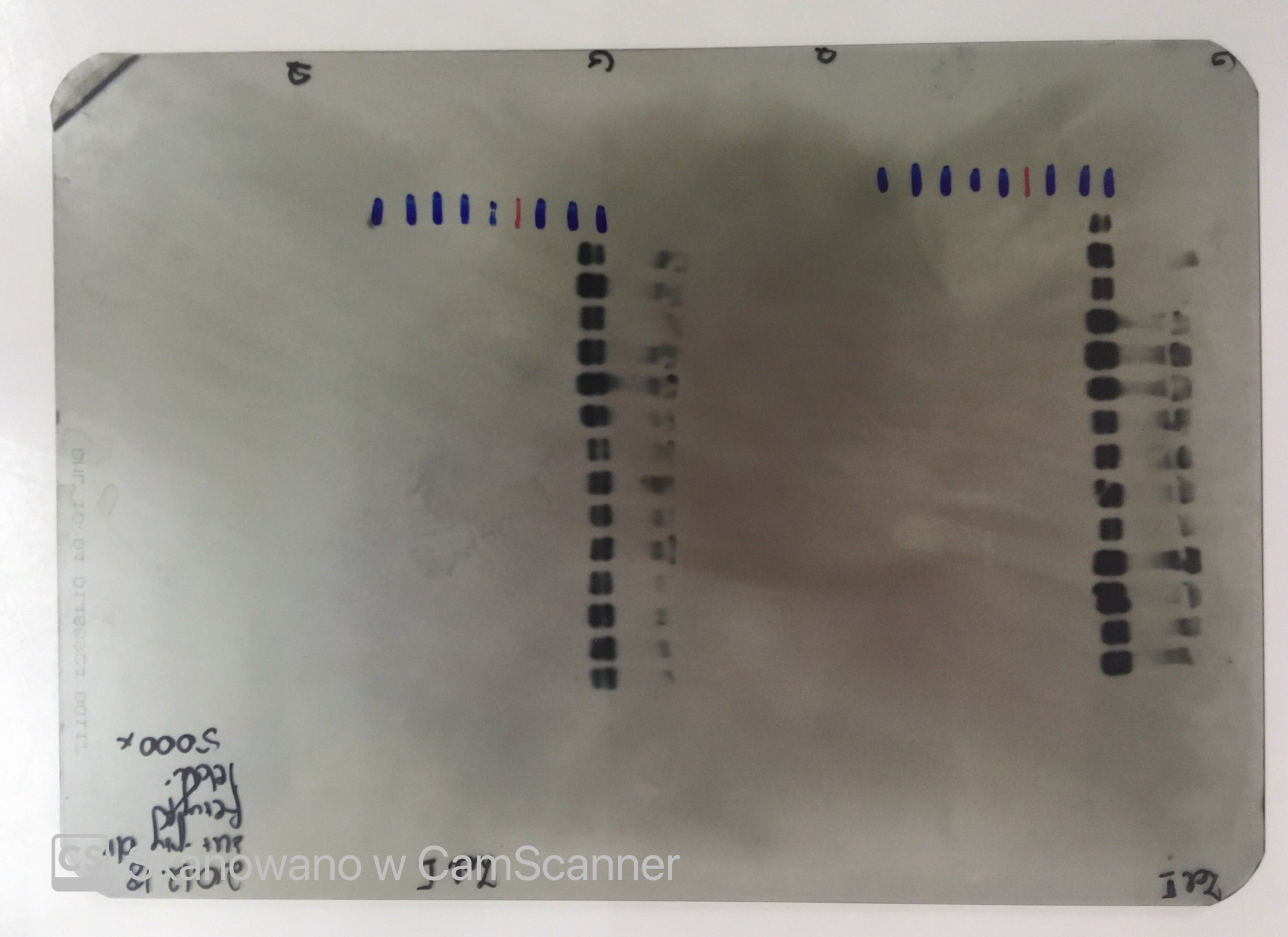

Supplement: Supplementary file 4 — Supplementary Material 4 [file 12917_2024_4071_MOESM4_ESM.jpg]
